# Can bone apposition predict the retention force of a femoral stem? An experimental weight-bearing hip-implant model in goats

**DOI:** 10.1186/s12891-015-0560-z

**Published:** 2015-04-28

**Authors:** Knut Harboe, Christian Lycke Ellingsen, Einar Sudmann, Nils Roar Gjerdet, Kjetil Søreide, Kari Indrekvam

**Affiliations:** Department of Orthopedic Surgery, Stavanger University Hospital, P.O. Box 8100, Stavanger, 4068 Norway; Department of Clinical Medicine, University of Bergen, Bergen, Norway; Department of Pathology, Stavanger University Hospital, Stavanger, Norway; Department of Clinical Dentistry, Biomaterials, University of Bergen, Bergen, Norway; Department of Gastrointestinal Surgery, Stavanger University Hospital, Stavanger, Norway; Kysthospitalet in Hagevik, Clinic of Orthopedic Surgery, Haukeland University Hospital, Bergen, Norway

**Keywords:** Uncemented, Total hip arthroplasty, Histological evaluation

## Abstract

**Background:**

The increasing incidence of prosthesis revision surgery in the Western world has led to an increased focus on the capacity for stem removal. We previously reported on a femoral stem implanted in goats with an approximate 15% reduction in retention force by drilling longitudinally orientated grooves on the side of the stem. In this current study, we aimed to histologically evaluate the bony apposition towards this stem and correlate this apposition with the pullout force.

**Methods:**

We analyzed the femora of 22 goats after stem removal. All stems remained in place for 6 months, and the goats were allowed regular loading of the hip during this time. For histological evaluation, all femora were immersed in EDTA and decalcified until sufficiently soft for standard technique preparation. We evaluated bone apposition, the presence of foreign particle debris and other factors. The apposition was evaluated with a scoring system based on semi-quantitative bone apposition in four quadrants. Kappa statistics were calculated for the score. We correlated the retention force with the amount of bone apposition.

**Results:**

The stem drilling was the only significant factor influencing the retention force (p = 0.020). The bone apposition Kappa score comparing poor and good apposition scores was fair (*κ* = 0.4, 95% CI 0.00–0.88). Signs of foreign body reaction were noted in 5 of 22 goats.

**Conclusions:**

Based on the current findings in an experimental goat model, it appears that the effect of drilling tissue/bone out of the longitudinal grooves has a more significant impact on the retention force required to remove the stem than the amount of bone apposition outside the stem grooves. This observation may be further explored in the research of stem designs that are potentially easier to remove.

## Background

Orthopedic prostheses have been implanted in an increasing number of patients and are considered one of the most successful and cost-effective surgical interventions available [1]. In the US alone, the number of total hip replacements and total knee replacements are expected to increase to 572,000 and 3.48 million, respectively, by year 2030. As a consequence, revision surgery is estimated to increase to 96,700 for hip revisions and 268,000 for knee during the same time period [2]. The increase in the revision burden within patients under 65 years is a particular challenge as these patients comprise approximately half of the number of all replaced and revised surgeries [3]. Optimal bonding between bone and implant is essential in orthopedic prosthesis surgery. However, the implants should simultaneously be removable, if indicated. Thus, increased research interest has focused on novel stem designs that can provide safe and durable joint replacement while facilitating easy removal when needed [4,5].

To this end, a novel femoral stem was designed to allow for easier removal without compromising retention properties [6]. The stem was coated with hydroxyapatite, which promotes bone ingrowth and bridges bone/implant gaps [7-9]. For evaluation in a weight-bearing model, the stem was implanted in goats. The goat experimental animal model demonstrates loading patterns and bone anatomy comparable to those of humans [10,11]. In previous experiments, a reduction in retention force by drilling out bone grown into longitudinal grooves on each side of the novel stem was demonstrated [6].

The aim of the current study was to evaluate whether the reduction of retention force could be explained by a difference in bone apposition towards the loaded hip stem rather than by the drilling itself.

## Methods

The experiments were performed in goats that were subjected to a total hip arthroplasty using a femoral stem as previously described [6]. A short summary of the methods used follows.

**Figure 1:**
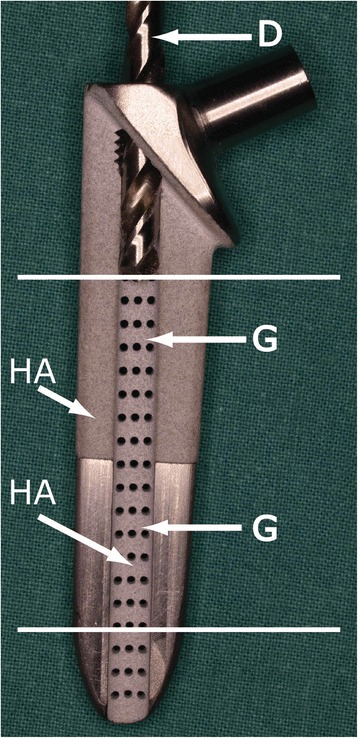
Novel stem design. Antero-posterior view. Hydroxyapatite-coated area (HA). Drill bit (D). Groove (G). White lines indicate levels of sections of bone specimens. Transverse canals are one millimeter in diameter.

Based on preoperative radiographic images of the goats’ femora, a novel stem was created that measured 70 mm in length, had a medial collar and was made of grade 5 Ti6Al4V. The stem had two semicircular longitudinal grooves connected with 69 canals, each 1 mm in diameter. Hydroxyapatite (HA) was applied over the blasted area (Figure 1). The coating was approximately 80 μm thick with a porosity of < 5% and approximately 67% crystallinity. A standard 17-mm total hip arthroplasty (THA) head designed for canine use and a corresponding cemented acetabular component were used.

The goats were operated on and received follow-up as outlined in our previous paper [6].

The animal study was approved by the Norwegian Animal Research Authority (Reference number 07/82783, 31.10.2007).

The goats were euthanized with a bolus of 2 g of pentobarbital administered intravenously. The left femur and ipsilateral portion of the pelvis were explanted with the prosthetic components in situ. The specimens were maintained on ice until being radiographed and CT scanned the same day and biomechanically assessed the following morning.

In total, 23 femora were eligible for inclusion in the biomechanical testing. The distal portion of the femur was embedded in acrylic cement (Meliodent, Heraeus-Kulzer GmbH, Hanau, Germany) in a custom-made steel cylinder that allowed vertical alignment and fastening to the pullout test machine. In order to screen for any gross instability of the construct, the femoral head and the greater trochanter were loaded at a right angle to the long axis with a low force (4 N) while recording the relative movement of the head (spring loaded force applicator and differential displacement recorder).

**Figure 2:**
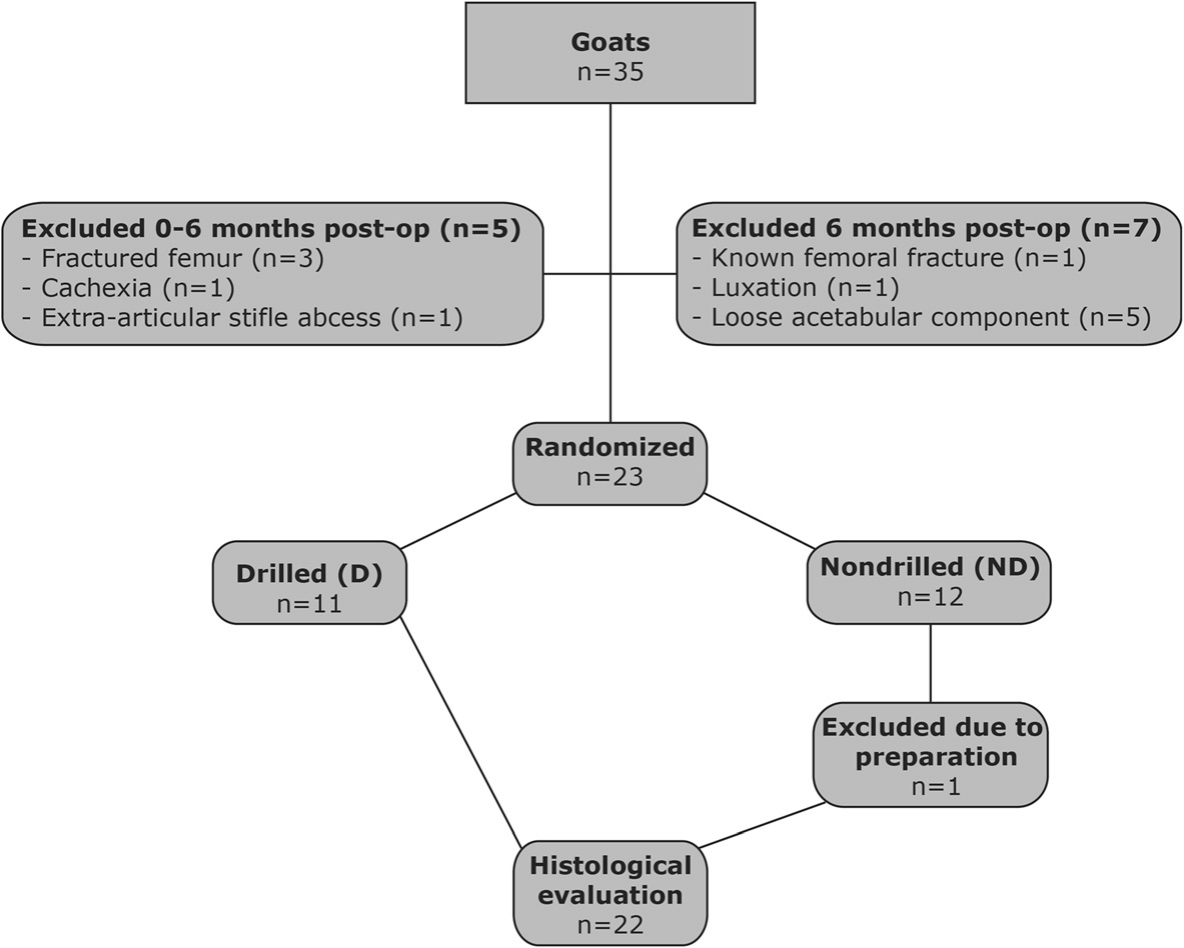
Flow chart of study. Allocations in groups and reasons for exclusion from the experiments.

To assess the effect of the longitudinal grooves anchoring to the surrounding bone, the femora with the implant in situ were randomized by coin toss into either the group subjected to stem pullout with drilling using a 4.5-mm drill-bit (Figure 1) to remove all of the tissues in the grooves (D-group) or the group without drilling (ND-group) (Figure 2).

A uniaxial test machine was used for the stem pullout (MTS 810, Minneapolis, Minnesota, USA). After the biomechanical testing, all stems were photographed to document the bony residue on the implants.

### Histology

After biomechanical testing, the included femora were fixed in 4% neutral buffered formalin. After complete fixation, two transversal slices, each approximately 4 mm thick, were cut from the proximal portion of all femora with a bone saw. The proximal slice was obtained from the level of the medial portion of the collar of the stem. The distal slice was obtained from the level corresponding to the last 1 cm of the stem (Figure 1). These slices were then decalcified with EDTA demineralizing solution (1000 ml 4% unbuffered formalin, 75 g EDTA, 14 g NaOH). We preferred decalcification with EDTA instead of nitric acid to ensure gentle tissue handling, although this process required a considerably longer time. The EDTA solution was changed every week, and the canisters with the bone samples were maintained on a rotating platform during the entire period. The bone samples were tested with a needle at every exchange of EDTA to ensure the appropriate level of decalcification. The decalcification process took approximately three months. The slices were then embedded in paraffin that was cut into 2–3 μm slices and stained with standard hematoxylin, erythrosine and saffron (HES).

In addition, we attempted to embed material from one goat in epoxy resin based on Hagen’s method [12]. In our hands, the histological quality was inferior to the typical method, so we chose not to continue with the epoxy embedding; this goat was excluded from the evaluation.

### Histological evaluation

**Figure 3:**
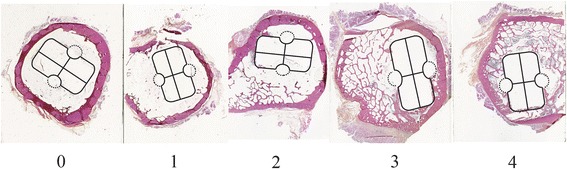
Example of evaluation of score. The score corresponds to the number of sectors with a visible reaction to the implant with apposition. Distortion of the stem outline is due to the processing of histological sections.

Two investigators (KH and CLE) independently evaluated all samples. For each parameter, a semi-quantitative score was provided as follows. The sample was divided into 4 sectors, excluding the grooves. In each sector, the bony response (i.e., bone apposition to the outline of the stem) was evaluated. If a bony response was noted, the sector was given one point. Hence, a sample could receive 0–4 points. In total, 2 samples were assessed from each goat, thereby providing a score ranging from 0–8 points (Figure 3). In the case of discrepancy, the specimen was jointly examined to reach consensus. It was not possible to directly study the interface between the bone and the prosthesis given that the prostheses were pulled out during the biomechanical testing.

Other aspects evaluated included the presence of leukocytes and macrophages, suggesting infection or reaction to coating or other foreign body. In addition, osteomyelitis was diagnosed based on bone necrosis outside the prosthetic cavity and the presence of neutrophilic granulocytes or microorganisms.

All of the slides were evaluated for possible birefringent foreign bodies using polarizing filters on the microscope (Olympus BX51 with U-Ant and U-Pot filters, Olympus Corp, Japan).

### Statistics

Correlations were analyzed using Spearman’s rank correlation, and the comparison of scores within the group as a total was assessed using the Mann–Whitney *U* test. Interrater reliability was analyzed using Kappa statistics. The score was also analyzed according to division into poor (0–3 points) and good (4–8 points) apposition groups. Descriptive analyses were performed with numbers presented as the median and range. Multiple regression analysis compared the drilled status and bone apposition score.

SPSS 21.0 (SPSS Inc., Chicago, IL) was used for statistical analysis. All tests were 2-tailed, and the significance level set at p < 0.05.

## Results

The experimental design and specimens included in the evaluation are presented in Figure 2.

The radiographs and CT scans did not revel any sign of osteolysis or implant instability. There were not documented large bony residues on the stems with visual inspection. There was not observed any gross instability of the stems. The mean retention force in the drilled group was 1526 N and in the non-drilled group 2033 N (p = 0.028).

The interrater agreement of the bone apposition score including all scores was analyzed with a Kappa value of 0.05 (95% CI 0.00–0.31) and 18% agreement (4 of 22). With a dichotomized score, the Kappa value increased to 0.40 (95% CI 0.00–0.88), and the percentage of agreement was 82.6% (19 of 22).

**Figure 4:**
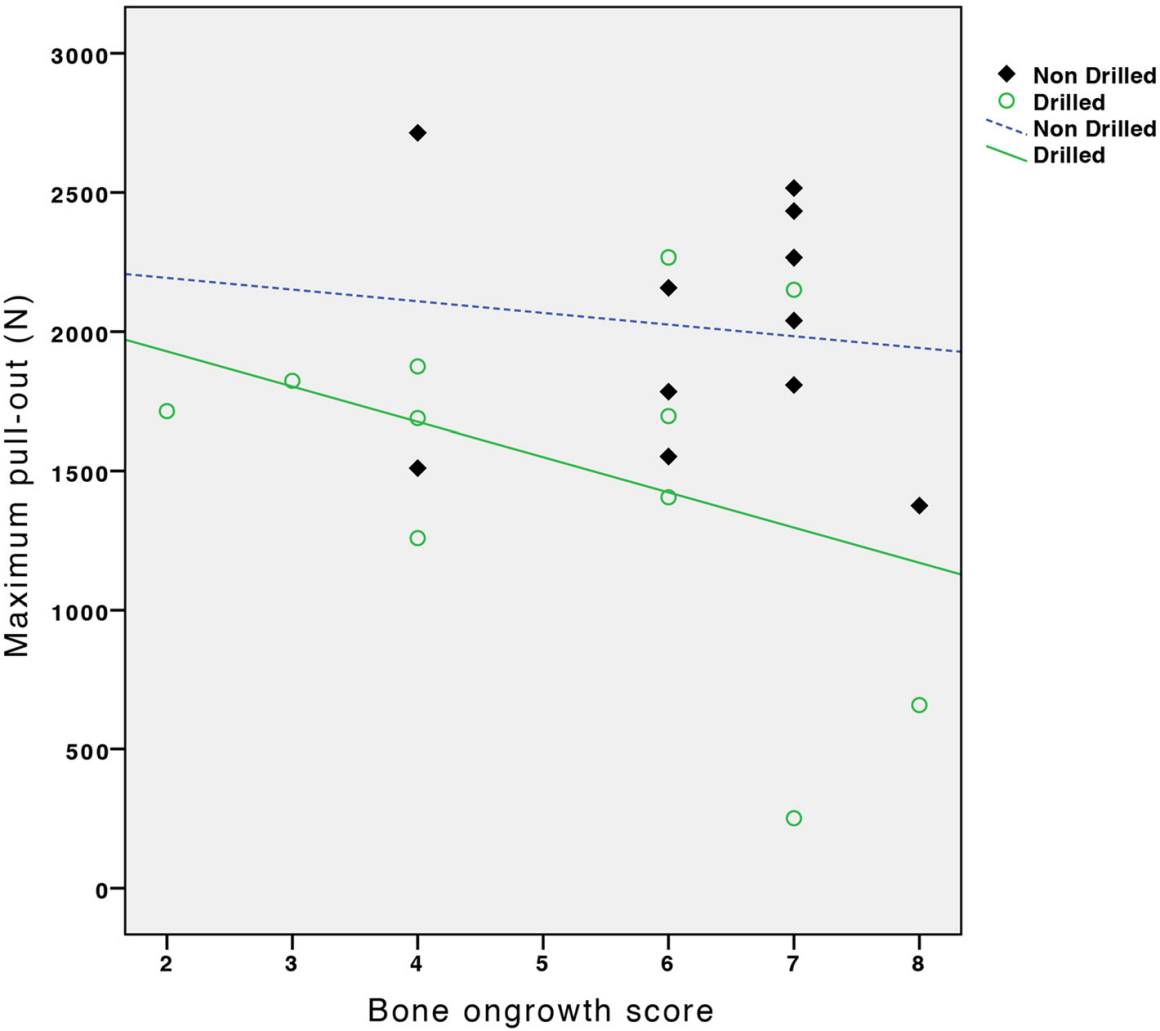
Bone apposition and pullout force. Scatterplot of sum of bone apposition scores on two levels and maximum pullout force (N). No significant difference was noted between the groups.

The median bone apposition scores for drilled and non-drilled specimens were not significantly different (median score 6, range 2 – 8, p = 0.17, Mann–Whitney). No significant correlation was observed between the sum of the two level scores and maximum pullout force (Spearman’s rho r_s_ = -0.17, p = 0.94) (Figure 4). For the drilled and non-drilled specimens, no significant correlation was demonstrated for bone apposition (drilled: r_s_ = -0.29, p = 0.38; non-drilled: r_s_ = -0.053, p = 0.88) (Figure 4).

Using multiple regression analysis, the drilling of the stem was the only factor that significantly affected the retention force of the stem (p = 0.020).

When examining the extent of bone apposition on the proximal and distal portions of the stem outside the groove, no significant correlation was noted between the groups, but a significant difference between the proximal and distal portion was noted when the group was considered as a total (median proximal score 4 (1–4), median distal score 2 (0–4), (Z = -2.75, p = 0.006, Wilcoxon Signed Rank)).

We did not observe any cases with greater than expected inflammation in the healing bone tissue or with a severity suggestive of osteomyelitis or peri-prosthetic infection. In 5 goats, we observed an infiltrate dominated by macrophages, which is potentially suggestive of a foreign body (granulomatous) reaction. No birefringent bodies were visible in these infiltrates. Small amounts of birefringent foreign bodies were noted in 8 of 22 goats. No visible sign of heat effect on the tissue from drilling was noted (basophilic homogenization of collagen and elongated nuclei).

## Discussion

The need for long lasting implants with a predictable revision setting is important for the future use of total hip replacements. Our new design exhibits a 15% reduction in retention forces after drilling the longitudinal grooves [6], and the possible confounding factor of bone apposition outside the groove must be evaluated. In this experimental model, we observed no significant difference in the bone apposition between drilled and non-drilled femora. In addition, we did not observe a correlation between bone apposition and retention force with the methods and scoring used. We believe this finding indicates an increased importance of the area in the longitudinal grooves for retention of the stem compared with the importance of adequate bone apposition to the stem surface itself. However, several aspects and some limitations to this observation are discussed and deserve further elaboration.

The traditional and natural evaluation of bone/implant interfaces occurs with the implant in situ. Given that the implants were removed using the pullout test, which is necessary to evaluate retention force, the evaluation of the implant in situ was not feasible in the current study. Our scoring method is potentially influenced by the distortion of bone/implant interface during stem removal. We previously reported that the removed stems exhibited minimal residual bone attached [6]. However, this observation does not eliminate the possibility that some bone could have been lost during section processing.

No established scoring systems are available for histological evaluation of bone apposition in femora after removing the implants. Consequently, we developed a pragmatic scoring system for a semi-quantitative evaluation of the bone apposition. The interrater reliability analysis demonstrated poor agreement [13] when using all 9 categories, which may be expected. However, the agreement increased to fair for dichotomized subgroups (“poor” and “good” ingrowth) as evidenced by Kappa values, and greater than 2/3 of cases received similar scores from both scorers. Notably, the score attempts to provide a somewhat more objective view of bone apposition, which we believe to be better than subjective interpretation by the investigator. However, we recognize that this method may have under- or overvalued the amount and quality of bone apposition given that a “gold standard” for bone apposition was not available for comparisons.

The evaluation of bone apposition in the samples did not correlate with the pullout strength of the implants. Our model exhibits an even distribution in retention force between the drilled and non-drilled groups, and the drilling of the stems was the only significant factor. This result confirms the findings of a previous study on the same stem that demonstrated an approximately 15% reduction in retention force after drilling the longitudinal grooves. In a human application of a stem incorporating these longitudinal grooves, it is of importance that the area outside the grooves does not contribute too much to the retention force. We do not encourage using drilling along conventional stems without such grooves.

The loading patterns of total hip replacements in goats have been studied previously [10,11] and were found to adequately simulate the human hip. The exclusion of animals at 6 months (Figure 2) was due to uncertain loading conditions. We chose goat as our model given that the animal is easy to handle, facilitates a large implant in the femoral canal and is very active, thereby potentially replicating human conditions better than small animal models. However, activity is also a major challenge given the strain placed on this loaded implant. This strain can partly explain the large percentage of animals excluded in this study. In one study [14], the fact that sheep has little spongy bone in the acetabular socket was used as a model for an augment of fixation of an acetabular component in sclerotic bone. This notion was not observed in our goat model, but we cannot eliminate the possibility that the spongy bone is of poorer quality than in humans.

The interface between bone and femoral implants in large animal models has been evaluated biomechanically in combination with histological, electron microscopy, and image analyses. Søballe [15] investigated stable and unstable implants in dogs and reported that HA bridges the gaps in the interface. The fibrous membrane was analyzed after push-out measurements. The implant/bone contact was not quantified in the push-out samples. Borsari [16] implanted TiAl6V4 rods coated with commercially pure titanium with and without hydroxyapatite (HA) coating in sheep. The observation time was three months. The implants were not loaded. After the push-out test, the contralateral side with the rod in situ was histologically evaluated, and the specimens with the implant removed were assessed by scanning electron microscopy. Analysis of the breakage of the interface among bone/HA/titanium exhibited fracture within the bone and not at the HA/bone interface. The bone/implant interface was not quantified. In our study, the breakage appears to occur at the HA/bone interface, and the implant was loaded. In other studies, a bone/implant contact model was used in sheep and goats. Biemond [17] tested E-beam (additive production method)-produced implants in goats and suggested a method for measuring the bone/implant contact in sectors as well as maximum depth of bone ingrowth. No biomechanical evaluation was performed after implantation. Our scoring system uses bone sectors and includes a biomechanical correlation. Coathup and Kalia et al. [18,19] investigated various surface treatments of mass tumor prosthesis in sheep. All analyses were performed on the bone/implant interface with the implant in situ and included finite element analyses. The biomechanical correlation was not assessed in contrast to our model. Later, Kalia [20] analyzed the interface of HA-coated acetabular components with or without a layer of fibrin glue embedded in bone marrow-derived stromal cells in goats. Again, the implant/bone interface contact was quantified with the implant in situ. A biomechanical evaluation was not performed. These studies demonstrate that solid osteointegration is expected with titanium implants with a surface coating similar to the one used in the current study. Most of the studies were performed with the implant in situ in contrast to the current study, wherein the implants were removed. The different studies are summarized in Table 1.

**Table 1:** **Overview over papers involving studies of bone/implant interface**

**Author**	**Animal**	**Outcome measure**	**Surface treatment**	**Implant**	**Loading**
Søballe	Canine	Biomechanical	HA	Stable/unstable screw	Loaded
Borsari	Ovine	Biomechanical Histology	HA, cp-Ti	Rods	Unloaded
Biemond	Caprine	Histology	TiAl6V4	Plugs	Unloaded
Coathup	Ovine	Histology + FEA	HA	Plates/prosthesis	Loaded
Kalia	Caprine	Histology	TiAl6V4 + BMSC	Acetabular cup	Loaded
Harboe	Caprine	Biomechanical Histology	HA	Femoral stem	Loaded

HA = Hydroxyapatite. Cp-Ti = commercially pure titanium. TiAl6V4 = Implant alloy consisting of 90% titanium, 6% aluminum and 4% vanadium. BMSC = Bone marrow stromal cells.

We hypothesized that increased bone apposition would increase the retention force of the implant. This hypothesis was not demonstrated, but the score does provide a semi-quantitative impression of the apposition of bone towards the stem. This notion is evident in the significant difference in the apposition scores in the proximal and distal areas of the stem. One would expect this finding given that spongy bone is more prevalent in the proximal portion of the femur. Enhanced bone apposition toward roughened surfaces is also evident compared with turned surfaces [21], which also supports the higher score in the proximal area of the stem.

## Conclusions

In conclusion, the novel stem exhibits good bone apposition and confirms our previous findings. Drilling reduces the retention force, and difference in bone apposition does not confound this result. Our pragmatic approach to evaluate the bone/implant interface of removed implants can be useful in the absence of a validated scoring method.
